# Task Irrelevant External Cues Can Influence Language Selection in Voluntary Object Naming: Evidence from Hindi-English Bilinguals

**DOI:** 10.1371/journal.pone.0169284

**Published:** 2017-01-12

**Authors:** Divya Bhatia, Seema Gorur Prasad, Kaushik Sake, Ramesh Kumar Mishra

**Affiliations:** 1 Maharishi Dayanand University, Rohtak, India; 2 Center for Neural and Cognitive Sciences, University of Hyderabad, Hyderabad, India; Leiden University, NETHERLANDS

## Abstract

We examined if external cues such as other agents' actions can influence the choice of language during voluntary and cued object naming in bilinguals in three experiments. Hindi–English bilinguals first saw a cartoon waving at a color patch. They were then asked to either name a picture in the language of their choice (voluntary block) or to name in the instructed language (cued block). The colors waved at by the cartoon were also the colors used as language cues (Hindi or English). We compared the influence of the cartoon’s choice of color on naming when speakers had to indicate their choice explicitly before naming (Experiment 1) as opposed to when they named directly on seeing the pictures (Experiment 2 and 3). Results showed that participants chose the language indicated by the cartoon greater number of times (Experiment 1 and 3). Speakers also switched significantly to the language primed by the cartoon greater number of times (Experiment 1 and 2). These results suggest that choices leading to voluntary action, as in the case of object naming can be influenced significantly by external non-linguistic cues. Importantly, these symbolic influences can work even when other agents are merely indicating their choices and are not interlocutors in bilingual communication.

## Introduction

Linguistic communication happens in social scenarios where language choice of others can modulate our choice. Bilingual speaker’s language choice involves selecting words from the context-appropriate language while inhibiting the others [1]. Many times this choice depends on the person to whom the speech is addressed. Language selection is influenced by both top-down and bottom-up factors [2],[3],[4]. Apart from direct interlocutors, language choice is often driven by other agents' choices and actions. For example, one tends to select a language to speak if this language was also chosen by someone else in that context. Visual cues that are linked to languages also prime language selection [2]. Here we examined if a cartoon’s choice of a language influences how bilingual speakers select a language to name an object. We investigated if external influences in the form of other agent’s actions modulate voluntary switching during object naming. The cartoon which waved at the color patches was not a conversation partner and did not name objects itself. These colors were also used as response cues for language choice during the naming task. We predicted that such an association between the color and the response would influence how bilingual speakers select the language when naming was voluntary and also when it was cued.

Voluntarily choosing a language to name an object on successive trials involves switching. Bilingual speakers switch between languages during their natural interaction. While switching to a cue does not call for top-down decision making, voluntarily switching involves both top- down and bottom-up considerations. These may include switching history, language preference linked to dominance, and bottom-up cues related to stimulus [5],[6],[7],[8]. While many studies have examined how bilinguals name objects in cued naming paradigms [9],[10],[11],[12],[13],[14],[15], not much is known about how bilinguals voluntarily choose one language over another when given a free choice. Further, no previous study has shown if actions of others should induce voluntary switching behavior in a non-discourse scenario, whereas it has been shown that speakers align with their interlocutors during active conversations [16]. Here we investigated if cues such as other agent’s actions should influence how bilingual speakers voluntarily choose the language to name an object. Thus we examined the dynamic interactions between top-down and bottom-up mechanisms that influence choice behavior during object naming in bilinguals.

Gollan and Ferreria (2009) [6] examined if language dominance influenced patterns of switching in bilingual object naming when the task was voluntary. Speakers were encouraged to select freely the language they wished to use for naming (Experiment 1). They observed robust switch costs even when participants chose the language voluntarily. Additionally, balanced bilinguals exhibited higher switch rate compared to English-dominant bilinguals, probably because the names of the pictures were more accessible to such bilinguals. This suggests that the switching pattern is influenced by the lexical accessibility of the pictures. In Experiment 2, the speakers were asked to choose both languages approximately an equal number of times. This time switch rate was higher compared to Experiment 1. Participants switched more often, in this case, to meet the task requirement of maintaining the balance between the two languages. Surprisingly, in this experiment, stay costs were observed instead of the usual switch costs. However, in this study, there were no external primes that could modulate the voluntary choices of the speakers.

In another study, Gollan et al. (2014) [7] compared voluntary switching in both linguistic and non-linguistic task in three experiments. In the linguistic task, participants were presented with pictures of common objects and were asked to name the picture in either L1 or L2. The instruction was to name using "whatever language comes to their mind" thereby placing no constraints on language selection. In the non-linguistic task, participants were presented with a set of digits and were asked to either "read aloud the number formed by the digits" or "add the digits and say the sum out loud." In Experiment 1, none of the stimuli were repeated, and a voluntary advantage (faster naming latencies on voluntary trials) was found only for the non-linguistic task but not for the picture-naming task. There were no differences in switch costs between voluntary and cued trials. In Experiment 2, pictures to be named were frequently repeated and the voluntary advantage was observed for both linguistic and non-linguistic tasks. The switch rate and switch costs on voluntary trials were found to be lower compared to cued trials, for both picture naming and the non-linguistic task. Thus, switch costs were reduced during voluntary naming compared to cued switching in Experiment 2.

These findings suggest that lexical accessibility of the stimuli modulated the control mechanisms during voluntary naming. This pattern of results has been more recently shown by Kleinman and Gollan (2016) [5] where cost-free switches were observed in a repeated picture naming task. Participants were asked to name repeatedly a small set of pictures in the easiest language, a phenomenon the authors refer to as “bottom-up” switching. “Bottom-up” switching is said to be different from “top-down” switching in which the participants are forced to shift between languages (like in cued switching). The authors found that participants engaged in “bottom-up” switching incurred no switch costs as their switches were driven by the lexical accessibility of the pictures. In another study, Gross and Kaushanskaya (2015) [8] studied bilingual children on a voluntary naming task. It was found that bilingual children switched to the non-dominant language only while naming highly accessible items. More difficult items were named in the dominant language. The above-discussed evidence suggests that factors related to individual difference such as language dominance or proficiency may influence the choice of language during voluntary naming. Further, switch costs observed during language switching is related to language dominance of the participants as well as the lexical accessibility of the pictures to be named. Here we explored if this process is also influenced by other task-irrelevant external events such as someone’s perceived actions.

Since language production happens within a socio-linguistic context and amidst the presence of many other speakers and cues, it is natural to expect that decisions about language choice in bilinguals are influenced by such factors. Even if the language choice is voluntary and one has full control over the choice to be made, speakers are sensitive to environmental constraints. More recently, bilingual processing theories have emphasized the importance of contextual information on language control [2]. The adaptive control hypothesis [2] proposes that bilinguals, particularly those who live in a mixed language environment, develop a high level of sensitivity to environmental cues that are related to languages. That is, such bilinguals need to adapt flexibly to the changing demands of the languages in conversational contexts. Language cues can restrict activation of lexicons and facilitate language control. Therefore, the very nature of bilingual interactive communication demands that bilinguals be sensitive to such changes in the environment. Most previous studies on bilingual language control have used language cues that are task relevant to induce context in switching tasks (e.g. speak English when the cue is red vs. in Spanish when it is blue), but it is not known if language control mechanisms are affected when such explicit cues are not used.

Peeters, Runnqvist, Bertrand and Grainger (2014) [17] tested whether switch costs observed in cued switching studies is an artifact of the explicit language cues used in such studies. In this study, participants were asked to name pictures in one language, either French or English, within a block. Before the naming phase on each trial, participants had to perform a judgment task on a printed word (written in French/English). The authors observed that participants were slower naming pictures in French following a judgment task on an English word compared to a French word. No such costs were incurred during English naming. Thus, asymmetrical switch costs were observed between comprehension and production, even though the production task did not involve any switching. Similarly, Gambi and Hartsuiker (2016) [18] tested bilinguals on a joint switching task in which two participants took turns naming pictures. One of the participants (“non-switching participant”) always named pictures in Dutch. The other participant (“switching participant”) was asked to name pictures voluntarily in either of the languages (Dutch or English). It was found that non-switching participants named the pictures in Dutch slowly when the switching participants had named the previous picture in English rather than in Dutch. In both these studies, participants who were asked to name in one particular language and had no instruction to switch nonetheless incurred switch costs due to external cues. These studies provide evidence that cues that are not explicitly linked to languages can still activate lexical representations and influence the language control mechanisms.

In this study, we examined if a cartoon’s action of indicating a color would influence how a bilingual speaker chooses the language to name. More specifically, we wanted to know if such a bottom- up cue modulates switching when naming is voluntary. We draw this assumption from the rationale that bilingual speakers are, in general, sensitive to cues present in the environment that are linked to one of the languages in some manner [2]. These cues may include language or culture specific symbols and also possibly other agent’s choice of language. Language related cues available in the environment may trigger a bias in decision making in speakers which may influence their choice of language in a voluntary naming task. These cues may also override and modulate internally generated top down choices i.e. one wants to name in Hindi but names in English since there is a cue in the environment that biases the decision towards English. In contrast to psycholinguistics, substantial number of experiments in other areas of cognitive psychology [19],[20],[21],[22],[23] with simple visuomotor tasks have shown that both subliminal and explicit primes can influence free choices of actions in a powerful way even when these actions are voluntary.

### Current study

In this study, we examined the influence of a cartoon's choice of a color on voluntary and cued naming tasks in bilinguals. We explored if choices made by the cartoon influence language selection of the speaker even when the cartoon itself does not name or behave as an interlocutor. Bilinguals should be sensitive towards such gestures of others in the environment so as to select the relevant language node dynamically. Since the bilinguals used in our study were proficient in both the languages, it is expected that they would be sensitive to the cartoon’s waving at the colors mapped to languages. One important methodological novelty of our design was that although the cartoon waved randomly at a color square representing the language to be selected for naming by the speakers, the cartoon did not speak the name itself. Thus, there was no action to be imitated by the speakers, and the influence was only very indirect. Further, the cartoon did not as such ‘choose' to perform any action since the color squares were only related to the response cues.

Experiment 1 included an explicit choice stage where participants were required to indicate their choice of language before naming. We also explored the influence of instruction type in this experiment. In voluntary switching studies, most experimenters instruct the participants to make their choices keeping a reasonable balance between the two alternatives [19]. It is possible that this may induce a top-down strategy as opposed to when no constraints are placed while choosing. The other important aspect of the voluntary task design is the presence or absence of an explicit choice phase. Arrington and Yates (2009) [21] had found that executive control measures correlated with only choice behavior but not with task performance measures. This shows that separate cognitive processes may govern task choice and task performance. This is relevant for our current question about voluntary language choice in bilinguals. In our case, we wondered if the cartoon’s choice along with biasing the speaker’s choice would also influence the resulting action? Results can differ depending on whether an explicit choice was elicited (“double registration paradigm” [20]) or direct response was sought. This is important for language production studies since it is possible that after explicitly indicating their language choice, speakers may change their plan because of several factors. We have examined this possibility in Experiment 2 and 3 which did not have a distinct choice stage. Participants were asked to name the pictures in “whatever language comes to their mind.” Experiment 2 and 3 differed in the duration of time given for naming an object on voluntary trials. Participants were given more time on voluntary trials in Experiment 3.

We expected the colors indicated by the cartoon to bias participants' choices. Previous studies have shown that context influences variables related to switching such as switch rate and switch costs [24]. Thus, the alignment of speakers' choice with the cartoon should influence their language switching. In all three experiments, we predicted that the percentage of task switches congruent with the cartoon would be higher than switches incongruent with the cartoon. Considering the role of dominance previously found in voluntary language switching, we predict that the speakers' language dominance would interact with the congruency in language selection. In both voluntary and cued blocks, we expected the percentage of incongruent errors to be higher as a result of the conflict between the cartoon's choice and the language cue/participant's choice. Our participants were highly proficient in both Hindi and English. However, they were dominant in English. Thus, we predicted that they would, in general, be faster in naming in English as this was the language used by them more often.

## Experiment 1

In this experiment, we examined if the cartoon’s waving towards a color influenced the free choice of speakers before they named the object on voluntary and cued trials. There was an explicit choice stage before each task response on voluntary trials. Using such a paradigm, we asked the participants to explicitly indicate their choice first (on voluntary trials) and then perform the action.

We also examined how task instruction may influence voluntary choice. Previous studies have mostly asked participants to keep a balance during the free choice trials so as to avoid any strategy [19],[20]. It is likely that when asked to maintain balance during free choice, the task may not remain purely voluntary as such since this instruction may have an additional top-down influence. Gollan and Ferreira (2009) [6] investigated how this instruction can affect voluntary task switching during bilingual object naming. It was observed that bilinguals switched more when there was an instruction to maintain balance. Therefore, we allowed speakers in one condition to maintain balance and in another condition, no constraint was placed on their language selection. We examined this issue in our design to see if the cartoon's influence changed with regard to the task instruction. We expected participants to go more with the cartoon's preferences when they operate under no constraint as opposed to when they have to choose with instruction in mind. That is because, when speakers do not have a top-down constraint of maintaining balance, it is likely that they will show higher sensitivity to the cartoon's behavior concerning their choices.

### Methods

#### Participants

Thirty-one bilinguals from the University of Hyderabad community (twenty-three male, eight female, Mean age = 22.9 years, SD = 2.6 years) participated in the main experiment. All the participants were native speakers of Hindi and had acquired English as a second language at school. None of the participants were multi-linguals. All bilinguals had acquired L2 (English) before the age of five (mean age of acquisition of English was 4.8 years (SD = 1.9 years). All the participants had studied in schools where the medium of instruction was English. Thus, the participants were early users of L2 and were dominant in L2 as per their current language uses (See Table 1). All the participants were students of the University of Hyderabad who had been staying there for an average of two years and voluntarily participated in the study. They also provided written consent for their participation. The study was approved by the Institutional Ethics Commitee of University of Hyderabad.

**Table 1:** Language and demographic data of the participants in Experiments 1–3.

*Experiment 1*	Mean	SD
Age (years)	22.9	2.6
Age of acquisition of L1 (years)	2.9	1.2
Age of acquisition of L2 (years)	4.8	1.9
Years of education in L1	10.5	3.7
Years of education in L2	13.8	5.2
Vocabulary test score (L2)	53%	14.22%
Semantic fluency score (L1)	11.05	2.67
Semantic fluency score (L2)	13.85**	2.89
***Experiment 2***	Mean	SD
Age (years)	23.48	1.5
Age of acquisition of L1 (years)	2.28	1.32
Age of acquisition of L2 (years)	4.7	2.95
Years of education in L1	3.05	4.93
Years of education in L2	15.33	4.65
Vocabulary test score (L2)	41.17	9.6
Semantic fluency score (L1)	11.12	2.43
Semantic fluency score (L2)	14.67**	3.19
***Experiment 3***	Mean	SD
Age (years)	22.17	4.57
Age of acquisition of L1 (years)	2.06	1.06
Age of acquisition of L2 (years)	4.08	2.28
Years of education in L1	6.1	4.84
Years of education in L2	14.3	3.94
Vocabulary test score (L2)	52.11%	12.4%
Semantic fluency score (L1)	9.6	3.2
Semantic fluency score (L1)	12.9**	2.9

Note: Significant differences between L1 and L2 fluency scores

**p < 0.001.

#### Control tasks

All the participants completed an online vocabulary test (WordORnot, Center for Reading Research, Ghent University) which was administered to test their proficiency in English. The test consisted of categorizing a string of letters as a "word" or a "non-word". The total score was the difference between the percentage of correct and incorrect responses. A correct response referred to accurately categorizing words and non-words. The average score of the participants on the vocabulary task was 53%, (SD = 14.22%). The researchers who developed the test estimate that for non-native speakers of English, a score of around 33% suggests high level of L2 proficiency. Thus, our participants were highly proficient in L2. We also administered a semantic fluency task to gain further evidence of language fluency of the participants in Hindi and English. In the semantic fluency test, participants were given two categories each in Hindi ("Vegetables", "Birds") and English ("Fruits", "Animals"). They were asked to produce as many words as they could in each category in one minute. The average number of words produced per language per minute was calculated. Participants had greater semantic fluency score in English (M = 13.85, SD = 2.89) than in Hindi (M = 11.05, SD = 2.67) (*p* < 0.001).

#### Stimuli

Two hundred black and white drawings measuring 300 x 300 pixels were selected from Snodgrass and Vanderwart (1980) [25] and other online sources such as Google Images (See S1 Appendix for the line drawings). Pictures with phonologically similar onset in Hindi or English and pictures having multiple names in Hindi or English were not considered. Pictures were first screened by the first author who is a highly proficient Hindi-English bilingual. Ten Hindi-English bilinguals (Mean age = 21.5 years, SD = 0.81 years) who did not participate in the main experiment, were asked to rate the pictures for their names and frequency of use in Hindi and English. These participants were asked to rate the pictures on a Likert Scale of 1–5 (1 = Do not agree at all with the given name, 5 = Highly agree with the given name) for both Hindi and English names. We selected pictures whose mean average was above 3.5 (out of 5) by more than 80% of the participants. Mean name agreement score for the pictures for English was 4.46 (SD = 0.39) while for Hindi it was 4.43 (SD = 0.26). There was no significant difference for the name agreement of these pictures in both the languages (*p* = 0.781). Participants also rated the frequency of use of the name of these pictures, on the Likert Scale (1 –Never used, 5 –Frequently used). The mean word frequency in English was 3.85 (SD = 0.67) while for Hindi it was 3.96 (SD = 0.57). These ratings between the languages did not differ significantly (*p* = 0.593).

Two colored squares (Red and Green) measuring 64 x 84 pixels each in size were used as language cues. The mapping between the colors and the languages was counter-balanced across the participants. A 1000 ms video of a cartoon of size 1280 x 720 pixels pointing towards a red or green square measuring 64 x 84 pixels each was played at the beginning of every trial. The cartoon used both its right and left hand to indicate the color squares positioned on both sides an equal number of times randomly. The gender of the cartoon was counter-balanced across the participants.

### Procedure

**Fig 1:**
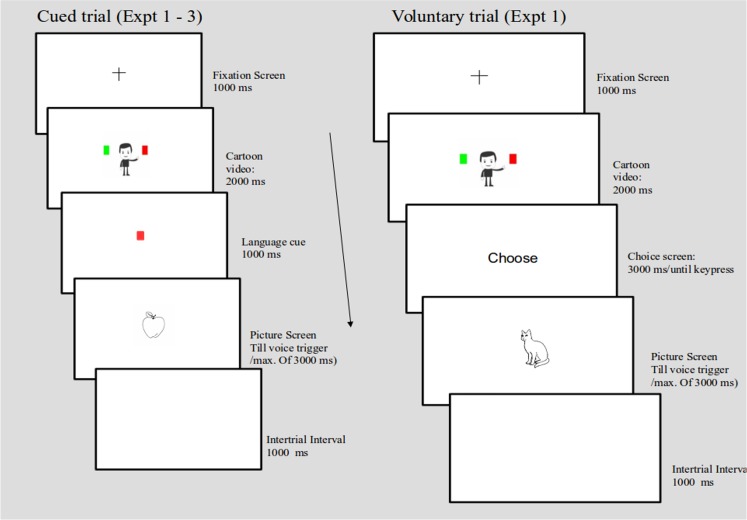
Sequence of events on cued trials (Experiment 1–3) and on voluntary trials (Experiment 1). The cued trial in this case refers to a congruent condition (cartoon waving towards “Red” matches the language cue “Red”).

**Fig 2:**
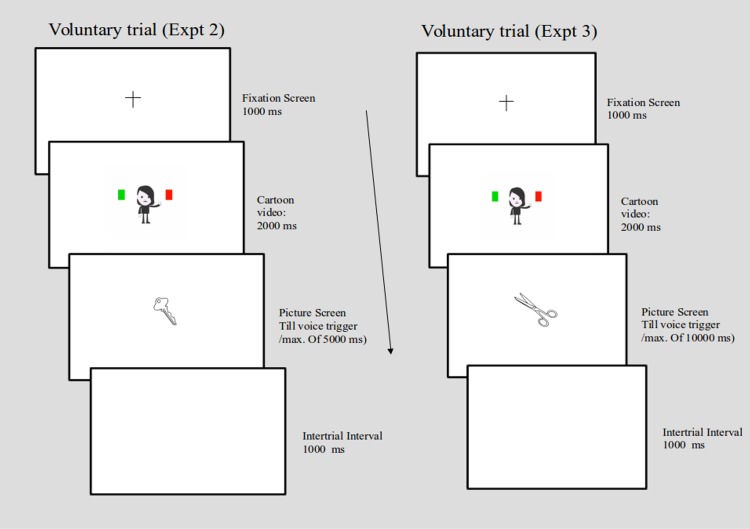
Sequence of events on voluntary trials in Experiment 2 and Experiment 3.

Before the main experiment, participants were familiarized with the stimuli pictures and their names in Hindi (L1) and English (L2). The line drawings were presented using DMDX (University of Arizona) version 5.1.1.3 with DirectX 9.0 on 19" DELL square monitor with 1280 x1024 pixel resolution and 60 Hz refresh rate. Participants were comfortably seated on a chair at a distance of 75 cm from the monitor. Response latencies were recorded by DMDX through a button press and voice trigger using an i-ball M-27 table microphone, respectively. Every trial started with a fixation cross at the center of the screen for 1000 ms, followed by a short video clip of the cartoon pointing towards one of the color squares positioned to the left and the right lasting for 2000ms (Figs 1 and 2). Then depending on the trial type either a language cue was presented for 1000 ms (cued block) or a choice screen was displayed till key-press or for a maximum of 3000 ms (voluntary block). In the cued block, after the language cue, the picture to be named was displayed for a maximum of 3000 ms. The picture disappeared as soon as the voice key registered a response. In the voluntary block, choices were registered using the index and middle finger of the right hand by pressing the keys "left-arrow" and "right-arrow" mapped to Hindi and English respectively which were marked with red or green colors. The mapping between the keys and the languages was counter-balanced across participants. If a choice was not made within 3000ms, the trial was aborted, and a message was displayed indicating the participants that they were too slow in choosing. Following the keypress, the object to be named was displayed till voice onset or a maximum of 3000 ms. The names of the pictures spoken by the participants throughout the experiment were recorded using audacity-win-2.0.5 and were exported for further analysis of errors. The participants were given 20 practice trials before the main experiment and the stimuli pictures in the practice trials were not repeated in the main experiment. An inter-trial interval was given for 1000 ms. Participants were asked to be fast and accurate in naming the objects. The whole experiment took about one hour including the control tasks. Participants were allowed to take a break in between the blocks.

Participants were told that the cartoon's waving at the colored squares is irrelevant for their task. This kind of instruction is very common for instance in cueing studies of spatial attention where a central arrow appears followed by targets. Participants in such experiments are explicitly told about the un-predictive nature of the arrow [26]. Similarly, we told the participants about the un-predictive nature of the cartoon’s waving for their task. Every participant repeated the experiment in two sessions. In one session, participants were told to "Choose each language approximately 50% of the times." This was called the "Maintain balance" condition. In another session, participants were told to choose "whatever language that came to their mind" [6]. This was called the "No constraint" condition. The two sessions were administered on two consecutive days.

### Design

Each session of the experiment (“Maintain balance” and “No constraint”) consisted of 160 trials divided into four blocks of 40 trials each. There were two blocks of voluntary trials and two blocks of cued trials. In each cued block, there were twenty congruent trials and twenty incongruent trials. On congruent trials, the language color cue matched with the language indicated by the cartoon. On incongruent trials, the language cue was different from that indicated by the cartoon. In voluntary Block, a trial was designated as "congruent" if the language chosen by the participant corresponded to the language indicated by the cartoon. Else, the trial was designated as "Incongruent". Therefore, congruency in the voluntary block was established (during analysis) with respect to the language (color) chosen and the language (color) waved at by the cartoon. Within each block, the trials were presented in a random order. The order of presentation of the blocks in each session was also randomized. The gender of the cartoon was counter-balanced across participants.

### Data analysis

Naming latencies were extracted for both cued and voluntary trials. 2.46% trials were excluded from the analysis where voice key was triggered due to non-verbal sounds or if participants didn't respond within 3000 ms while naming the pictures. We also excluded the trials with RTs less than 150 ms, resulting in the removal of 7.57% of trials from all conditions. Additionally, we removed 8.47% trials in which participants used the incorrect name for the pictures and were considered as error trials. These errors also consisted of trials on which the subjects chose a language but named in the other. Error analysis was further performed on these trials.

Data analysis was performed using mixed-effects models [27] using the lme4 package in R [28]. Visual inspection of residual plots did not reveal any deviations from normality. The analysis approach was similar to that used in other object naming studies [7],[29]. Separate models were created for choice percentages (only on voluntary trials as cued trials did not involve language selection), naming latencies and errors. The estimates and standard errors are given in the form of tables in the text. The full structure of the model along with random effect variances for the fixed effects are given in Tables A-G in S2 Appendix.

**Fig 3:**
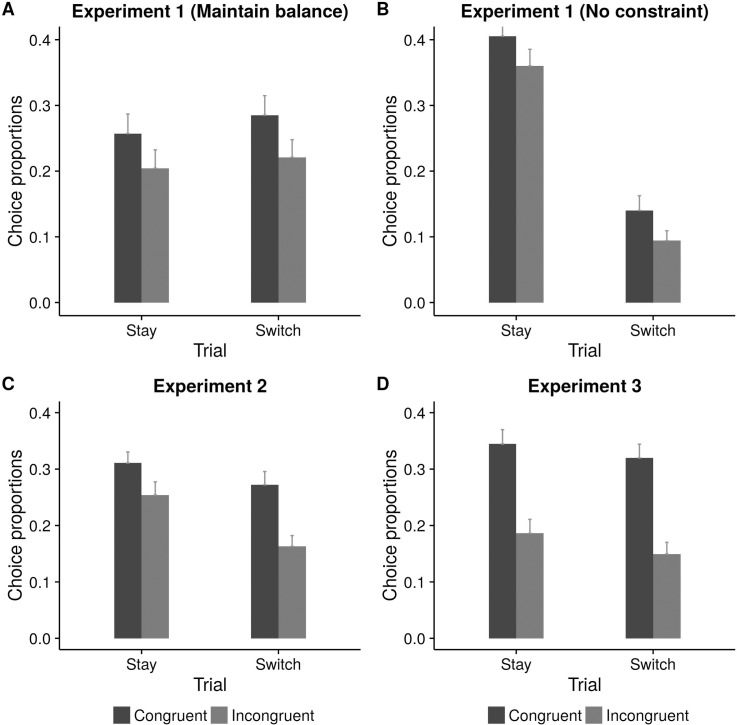
Proportion of congruent choices on stay and switch trials in “Maintain balance” condition (Experiment 1), “No constraint” condition (Experiment 1), Experiment 2 and Experiment 3.

**Fig 4:**
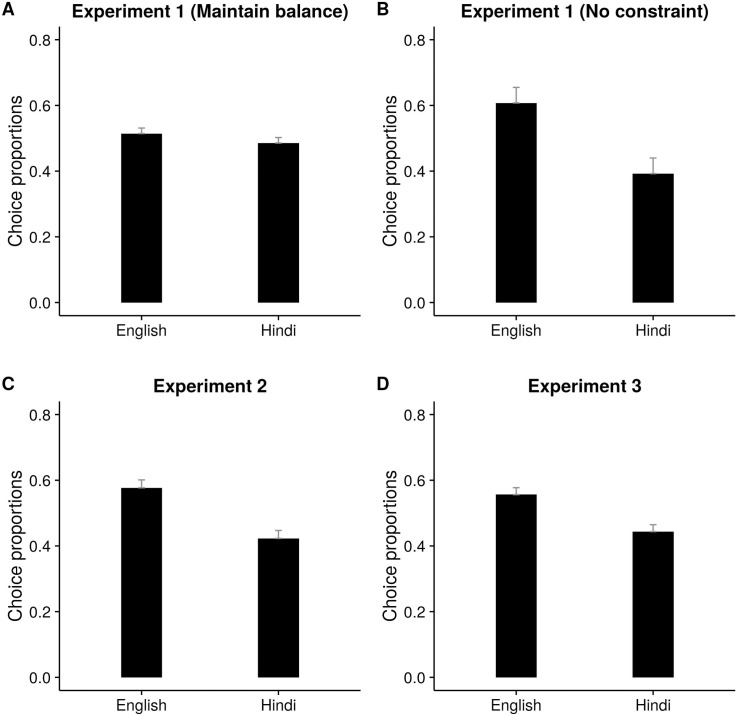
Proportion of choices as a function of language chosen in the voluntary block (Expt. 1–3)

**Choices:** Number of choices on voluntary trials was considered as the dependent measure in the model. Mixed-effects logistic regression was performed on the number of choices by specifying family as poisson and link log. Instruction type (“Maintain Balance”, “No constraint”), Language (Hindi, English), Congruency (Congruent, Incongruent) and trial type (stay, switch) were treated as fixed effects (Figs 3 and 4). On voluntary trials, congruency and trial type was established based on the classification of the responses after the experiment. Subjects were treated as random factors. Similar analysis was performed separately for trials in each instruction type.

**Fig 5:**
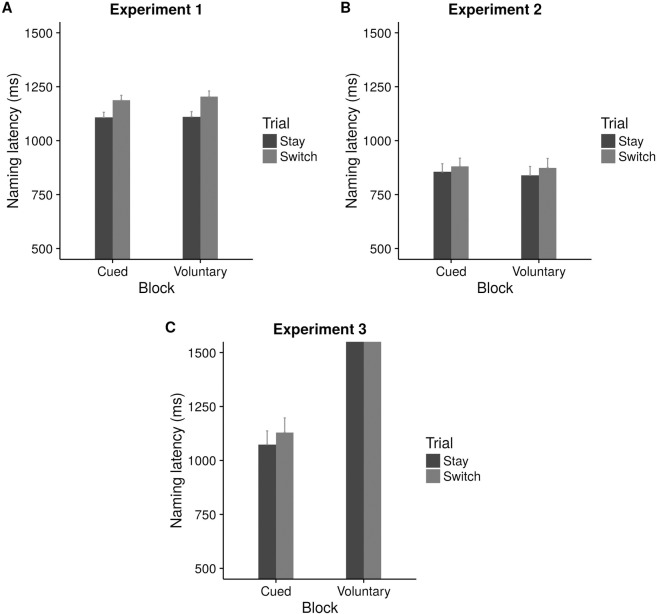
Naming latency on stay and switch trials on cued and voluntary blocks (Expt. 1–3).

**Naming latency:** Separate models were fitted for naming latency on cued and voluntary trials (Fig 5). A combined model could not be fitted as Instruction type was a fixed effect only for voluntary trials. Language, Congruency, and Trial type were common to both cued and voluntary trials. Subjects and items were treated as random factors.

**Errors:** Mixed efects regression models with family sepcified as binomial and link logist were fitted for Accuracy data. Separate models were created for errors in cued and voluntary block. Instruction type, Congruency, Language and Trial type were considered as fixed effects. Subjects and items were included as random factors.

For all the models, random slopes for fixed effects were included to give the maximal random effect structure justified by our sample [30]. All two-way and three-way interactions were included except in cases where the model could not converge. In accordance with previous analysis on large psycholinguistic data [7],[8], the statistical significance of effects was determined using t values. Absolute t values greater than or equal to 1.96 were considered statistically significant; absolute t values greater than or equal to 1.65 but less than 1.96 were taken to be marginally significant. Pairwise comparisons were done using “multcomp” package in R [31]

### Results

#### Language selection

In the voluntary block, type of instruction played a significant role (*t =* 7.57) in the choice of language of the participants. Importantly, participants selected a language that was congruent with the cartoon's choice of language significantly more often than the incongruent language but only in the “Maintain balance” condition (ß = -0.15, SE = 0.08, *t =* -1.98) (Table 2). English was chosen more often than Hindi (ß = -0.24, SE = 0.13, *t =* -1.74) in the “No constraint” condition (Fig 4). Another important result was the significant interaction between Trial type and Congruency (ß = -0.31, SE = 0.08, *t =* -4.06) which was seen only in the “No constraint condition” (ß = -046, SE = 0.16, *t =* -2.9). Cartoon's indication of language influenced the switching pattern of the bilinguals as indicated by a larger number of congruent switches than incongruent switches (*t =* 2.95). There was an interaction between Trial type and Instruction type (ß = -1.02, SE = 0.08, *t =* -13.04) (Fig 3) suggesting thatrepetition bias (that is, higher percentage of stay trials than switch trials) in language selection was higher in the “No constraint” condition (ß = -1.01, SE = 0.15, *t =* -6.65).

**Table 2:** Results on choices during the two instruction conditions derived from mixed-effect models. (Experiment 1).

	“Maintain balance” instruction				“No constraint” instruction			Across Instruction type	
Variable		Estimate	SE	*t*	Estimate	SE	*t*	Estimate	SE	*t*
Intercept		1.94	0.12	16.14*	2.59	0.12	20.73*	2.04	0.09	20.71*
Instruction								0.64	0.08	7.57*
Trial type		0.08	0.16	0.46	-1.01	0.15	-6.65*	0.05	0.10	0.47
Congruency		-0.15	0.08	-1.98*	-0.03	0.1	-0.33	0.02	0.09	0.19
Language		-0.03	0.07	-0.41	-0.24	0.13	-1.74^†^	-0.04	0.09	-0.49
Instruction * Trial type								-1.02	0.08	-13.04*
Instruction*Congruency								-0.03	0.09	-0.40
Instruction * Language								-0.22	0.09	-2.25*
Congruency*Trial type		-0.06	0.10	-0.6	-0.46	0.16	-2.9*	-0.31	0.08	-4.06*
Language*Trial Type		-0.02	0.1	-0.18	0.02	0.15	0.17	0.12	0.07	1.61 ^.^
Congruency* Language					-0.12	0.13	-0.93	-0.09	0.1	-0.9
Congruency*Language*Instruction					0.21	0.24	0.89	0.06	0.14	0.41

* *p* < 0.05

^†^*p* < 0.1.

#### Naming latency

Robust switch costs were observed in the cued block as indicated by a significance effect of trial type on naming latencies (ß = 78.76, SE = 29.99, *t =* 2.63) (Table 3). Speakers were also faster naming in English than in Hindi (ß = 95.81, SE = 29.32, *t =* 3.23). No effect of congruency was found (*t =* 0.56). None of the interactions were significant. For the voluntary block (Table 4), there was only an effect of trial type (ß = 111.19, SE = 41.48, *t =* 2.68) indicating costs while switching between two languages (Fig 5). However, this effect was seen only in the “Maintain balance”condition (ß = 98.23, SE = 38.62, *t =* 2.54). There was no evidence of asymmetric switch costs (as indicated by a language * trial type interaction) in the cued (*t =* 0.67) or the voluntary block (*t =* -0.04).

**Table 3:** Results on Naming latency in the cued block (Experiment 1).

Variable	Estimate	SE	*t*
Intercept	1060.52	34.98	30.32*
Trial type	78.76	29.99	2.63*
Congruency	22.13	39.47	0.56
Language	95.81	29.32	3.23*
Congruency*Trial type	-3.6	42.65	-0.08
Language*Trial Type	25.57	38.23	0.67
Congruency*Language	-24.95	40.66	-0.61
Congruency*Language*Trial type	-36.16	54.54	-0.66

* *p* < 0.05

^†^*p* < 0.1.

**Table 4:** Results on Naming latency in the voluntary block (Experiment 1).

		“Maintain balance” instruction				“No constraint” instruction				Across Instruction type		
Variable		Estimate		SE	*t*	Estimate		SE	*t*	Estimate		SE	*t*
Intercept		1120.76		45.26	24.76*	1097.05		39.61	27.69*	1105.32		38.69	28.56*
Instruction										-25.68		31.35	-0.82
Trial type		98.23		38.62	2.54*	28.39		40.29	0.48	111.19		41.48	2.68*
Congruency		4.42		40.62	0.11	6.04		26.99	0.22	19.26		38.89	0.49
Language		52.47		39.73	1.32	36.31		38.16	0.95	56.37		40.15	1.40
Instruction * Trial type										-28.34		48.98	-0.58^.^
Instruction*Congruency										-3.78		43.09	-0.09
Instruction * Language										2.43		44.59	0.05
Congruency*Trial type		-28.77		53.59	-0.54	-60.25		54.67	-1.02	-37.73		48.00	-0.79
Language*Trial Type		-2.5		51.20	-0.05	-7.74		53.99	-0.14	-1.75		46.42	-0.04
Language* Congruency		19.84		55.39	0.36	5.92		40.76	0.14	-9.09		48.43	-0.19
Trial type*Language*congruency		22.67		75.91	0.3	81.7		81.69	1.00	57.54		56.95	1.01
Trial type*Congruency*Instruction										-67.63		60.17	-1.12
Language*Congruency*Instruction										3.38		56.2	0.06
Trial type*Language*Instruction										3.74		59.91	0.06

^*^ p < 0.05

^†^p < 0.1.

#### Errors

Participants made greater number of errros in naming during switch trials than stay trials in the cued block (ß = 0.34, SE = 0.09, *t =* 3.6). There was no main effect of congruency (ß = 0.21, SE = 0.16, *t =* 1.34) or language (ß = -0.13, SE = 0.09, *t =* -1.43) in the cued block. In the voluntary block, Instruction type had a marginally significant effect on errors (ß = -0.34, SE = 0.18, *t =* -1.89). There were greater errors in the “Maintain balance” condition as opposed to the “No constraint” condition. There were also higher number of errors while naming in Hindi compared to English (ß = 0.35, SE = 0.10, *t =* 3.39). There was no main effect of congruency on errors (ß = -0.09, SE = 0.10, *t =* 0.88).

### Discussion

Speakers made more choices congruent with the cartoon when they were instructed to maintain balance in their choices. If the cartoon was pointing towards a colored square representing “Hindi”, participants were more likely to choose “Hindi” for naming the following object. The cartoon also influenced the switching pattern, especially in the “No constraint” condition. Participants were more likely to switch to a language indicated by the cartoon. Thus, the cartoon’s choice biased speakers' choice leading to an observable effect on language switching. These results clearly provide evidence for the influence of external primes on bilingual language selection.

Participants switched a greater number of times in the “Maintain balance” condition than in the “No constraint” condition. These results replicate Gollan and Ferreira (2009) [6] where they found higher switch rate when participants were asked to maintain balance while choosing languages as opposed to the condition where they were told to name with “whatever language comes to their mind.” There was less switching when there was no top-down constraint.

Participants incurred switch costs for both cued and voluntary blocks [32]. Naming latency was faster for English than Hindi. The error percentage was also lower in English. This could be attributed to the higher dominance level of our participants in L2. Gollan et al. (2014) [7] had similarly found faster response times and fewer errors on participants' dominant language during voluntary naming. However, we did not find any significant effects of congruency on naming latencies. Thus, the cartoon's influence was not evident on naming latency and was limited only to the choice of language

## Experiment 2

In Experiment 1, the cartoon’s influence was seen on language choice but not on the resulting action. This may suggest that speakers may still change the plan, or the naming process is independent of such influence. However, since we asked the participants to choose first and then name in Expt. 1, it is possible that this lead to the cartoon's influence being limited to the selection phase. It is also possible that participants were merely choosing the colored squares due to perceptual similarity with the squares being indicated by the cartoon. That is, speakers just went for the color the cartoon selected. Further, participants had no idea about the picture they were going to name although they had chosen a language for it. In contrast, in this experiment, we tested the influence of the cartoon when speakers did not make an explicit choice but named the objects directly. We asked if there will still be an influence of the cartoon’s choice of color on their naming preference and latency? Further, Experiment 1 showed that the cartoon's influence was evident in the switching mechanism of the participants only in the “No constraint” condition. Thus, in this experiment, we did not instruct the speakers to maintain any balance in their choice of language, to be parsimonious as this kind of a top-down instruction can induce strategies.

### Methods and participants

Twenty-seven Hindi English (Eighteen male, Nine female, Mean age = 23.48 years, SD = 1.5) bilinguals participated in the experiment. All the participants completed the control tasks as mentioned in Experiment 1 (Table 1). The mean vocabulary score was 41.17%. Semantic fluency scores in English (M = 14.67, SD = 3.19) were higher (*p* < 0.001) than Hindi (M = 11.12, SD = 2.43). The design was similar to that of Experiment 1. Two of the participants had taken part in Experiment 1.

### Procedure

The procedure was similar to Experiment 1 except for a change in the voluntary block. After the screen in which the cartoon indicated one of the squares, the line drawing of the object to be named was directly displayed for 5000 ms (Fig 2). In the cued block, the sequence of events remained the same as in Experiment 1.

### Data analysis

4.3% trials were excluded from the analysis where voice key was triggered due to non-verbal sounds or if participants didn't respond within the specified time while naming the pictures. 11.8% of trials were removed as error trials on which further analysis was performed. We analyzed the data following the method outlined in Experiment 1. Separate models were created for choices, naming latency and errors. Naming latency and errors were analyzed for cued and voluntary trials together and separately.

### Results

#### Language selection

English was chosen more often than Hindi (ß = -0.42, SE = 0.11, *t =* -3.59) (Table 5, Fig 4). Repetition bias in language selection was evident by the main effect of trial type (ß = -0.31, SE = 0.12, *t =* -2.5). Participants chose to “stay” with their selection from the previous trial than “switch”. The repetition bias was significant only in L2 (*t =* 4.08) but not in L1 (*t =* 0.66) as shown by a significant interaction between language and trial type (ß = 0.39, SE = 0.14, *t =* 2.75). Like in Experiment 1, there was a significant interaction (marginal) between Trial type and congruency (ß = -0.27, SE = 0.15, *t =* -1.85). Congruent switches were greater in number than incongruent switches (*t =* 4.92) suggesting that participants were more likely to switch to a language if it was also indicated by the cartoon (Fig 3).

**Table 5:** Results on choice data (Experiment 2).

Variable	Estimate	SE	*t*
Intercept	2.23	0.09	23.07*
Trial type	-0.31	0.12	-2.5*
Congruency	-0.13	0.09	-1.5
Language	-0.42	0.11	-3.59*
Congruency*Trial type	-0.27	0.15	-1.85^†^
Language*Trial Type	0.39	0.14	2.7*
Congruency*Language	0.04	0.15	0.24
Trial*Congruency*Language	-0.03	0.22	-0.15

* *p* < 0.05

^†^ *p* < 0.1.

#### Naming latency

No significant effect of block was observed (*t =* -0.06) indicating the absence of voluntary advantage in naming latency (Table 6). Cartoon's influence did not extend till naming latency as indicated by a non-significant effect of congruency (ß = 54.79, SE = 46.39, *t =* 1.18). There was no significant effect of Trial type either (*t =* 0.52) indicating an absence of switch costs across cued and voluntary blocks (Fig 5).

**Table 6:** Results on naming latency in cued and voluntary blocks (Experiment 2).

	Cued block			Voluntary block			Across blocks		
Variable		Estimate	SE	*t*	Estimate	SE	*t*	Estimate	SE	*t*
Intercept		860.69	49.4	17.42*	845.53	47.44	17.82*	851.32	48.96	17.39*
Block								-2.93	50.07	-0.06
Trial type		9.74	49.95	0.19	14.37	38.36	0.37	23.65	45.15	0.52
Congruency		42.09	51.09	0.82	18.94	36.43	0.52	54.79	46.4	1.18
Language		-33.59	51.77	-0.65	-7.6	39.28	-0.19	-22.43	42.72	-0.52
Trial type*Block								-13.79	55.98	-0.25
Language*Block								11.02	54.49	0.20
Congruency*Block								-45.62	56.74	-0.8
Trial type*Congruency		-46.91	70.66	-0.66	1.96	58.54	0.03	-76.26	59.62	-1.28
Congruency*Language		2.9	69.99	0.04	-60.54	58.76	-1.03	-24.38	56.86	-0.43
Trial type*Language		93.7	68.83	1.36	22.58	55.54	0.41	67.25	56.98	1.18
Trial type*Congruency*Block		-20.76	98.72	-0.21	89.41	87.37	1.02	99.27	68.3	1.45
Congruency*Languge*Block								-14.83	65.29	-0.23
Trial type*Language*Block								-31.18	65.95	-0.47
Trial type*Language*Congruency								42.58	65.59	0.65

* *p* < 0.05

^†^ *p* < 0.1.

#### Errors

Participants made greater number of errors on switch trials in the cued block compared to stay trials (ß = 0.53, SE = 0.24, *t =* 2.2). Errors were also significantly higher while naming in Hindi as opposed to English (ß = 1.7, SE = 0.21, *t =* 8.1) in the cued block. Congruency did not have a significant influence on errors (ß = 0.21, SE = 0.24, *t =* 0.86). No significant main effect of congruency (ß = -0.09, SE = 0.25, *t =* -0.35), language (ß = -0.13, SE = 0.24, *t =* -0.56) or trial type (ß = -0.35, SE = 0.24, *t =* -1.5) was found in the voluntary block.

### Discussion

Speakers' language switching was influenced by the cartoon’s choice of the color square, although this effect was only marginally significant. We found the characteristic switch costs in naming across cued and voluntary blocks. Similar to Experiment 1, we did not find an effect of the cartoon on naming latencies in both the blocks. We had hypothesized that introducing an explicit choice stage might have led to the absence of the cartoon's influence on naming latency in Experiment 1. The cartoon's actions did not modulate the speed of naming even though it modulated the switch pattern.

## Experiment 3

Both Experiment 1 and 2 suggest that the color cue influenced speakers' choice of language. This influence of the cartoon’s action led to higher congruency in choices but did not influence naming latency. Previously it has been shown that primes often do not influence voluntary action if there is a long delay. Agents probably bring in better top-down control for their choices when they get more time. We speculated that participants might have chosen the language along with the cartoon in Experiment 1 and 2 because there was less time to make a top-down decision. So, we wanted to test whether the availability of a longer decision time would influence the language selection pattern. To test this proposal, we increased the SOA between the cartoon’s action and speaker’s choice to 10,000 ms.

### Participants and stimuli

Twenty-three Hindi English bilinguals (Eleven male, Twelve female, Mean age = 22.17 years, SD = 4.57) participated in this experiment. The mean vocabulary score was 52.11%. Semantic fluency scores in English and Hindi were 12.1 and 9.6 respectively (See Table 1). Three of the participants had participated in Experiment 2 and four in Experiment 1. The participants who had already taken part in Expt. 1 or 2 did not differ significantly from the fresh participants in terms of age (fresh participants: 22.7 years vs repeated participants: 23 years, *p* = 0.9) or language proficiency measures: Vocabulary test (fresh: 51.2% vs repeated: 54.4%, *p* = 0.64), L1 semantic fluency score (fresh: 9.03 vs repeated: 11, *p* = 0.25) and L2 semanctic fluency score (fresh: 12.9 vs repeated: 14.4, *p* = 0.4)

### Procedure

The procedure was similar to Experiment 2 except for one minor change (Fig 3). The line drawing of the object to be named stayed for a total duration of 10, 000 ms on voluntary trials. The design and other aspects of the experiment remained similar to Experiment 2.

### Data analysis and results

Data analysis procedure was similar to Experiment 2. 2.03% trials were excluded from the analysis where voice key was triggered due to non-verbal sounds or if participants didn't respond within the specified time while naming the pictures. Naming latencies less than 150 ms resulting in the removal of 2.53% of trials from all conditions. There were 6.3% trials which were considered as errors.

#### Language selection

Participants chose the language indicated by the cartoon greater number of times than otherwise (ß = -0.53, SE = 0.09, *t =* -5.77) (Table 7, Fig 3). This effect was found for both L1 and L2 choices (*t =* 5.61 and *t =* 7.07, respectively) as indicated by a marginally significant interaction between language and congruency, (ß = 0.26, SE = 0.15, *t =* 1.79) (Fig 3). English was chosen greater number of times compared to Hindi (ß = -0.46, SE = 0.10, *t =* -4.5; Fig 4). There was a significant effect of trial type (ß = -0.35, SE = 0.10, *t =* -3.42) indicating repetition bias. This effect was found significant only in L2 selection (*t =* 3.55) as indicated by a significant interaction between language and trial type (ß = 0.59, SE = 0.12, *t =* 4.72).

**Table 7:** Results of mixed-effects modelling on language choice (Experiment 3).

Variable	Estimate	SE	*t*
Intercept	2.62	0.07	34.71*
Trial type	-0.35	0.10	-3.42*
Congruency	-0.53	0.09	-5.77*
Language	-0.46	0.10	-4.5*
Congruency*Trial type	-0.05	0.14	0.41
Language*Trial Type	0.59	0.12	4.72*
Congruency*Language	-0.26	0.15	1.79^†^
Trial type*Congruency*Language	-0.45	0.22	-2.07*

* *p* < 0.05

^†^ *p* < 0.1.

#### Naming performance

There was a marginal effect of congruency on naming latency (ß = 119.21, SE = 68.58, *t =* 1.74) (Table 8). Participants were faster naming on congruent trials compared to incongruent trials but only in the cued block (ß = 106.59, SE = 52.25, *t =* 2.04) and not the voluntary block (ß = 56.57, SE = 66.23, *t =* 0.85). Naming was significantly delayed in the voluntary block in comparison to the cued block (ß = 669.39, SE = 78.08, *t =* 8.57). Participants were also faster naming in English compared to Hindi (ß = 135.71, SE = 69.65, *t =* 1.95) but only in the cued block (ß = 138.93, SE = 65.48, *t =* 2.12). There was a significant interaction between congruency and trial type across blocks (ß = -152.61, SE = 81.77, *t =* -1.89). Post-hoc comparisons showed marginally significant switch costs were observed for incongruent trials (*t =* 1.65). A significant interaction between congruency and trial type was also observed in the cued block, (ß = -132.65, SE = 72.44, *t =* -1.8) indicating significant switch costs on congruent trials (*t =* -1.7) (Fig 4). Language and congruency interaction was also marginally significant in the cued block, (ß = -125.61, SE = 69.99, *t =* -1.79) showing faster naming latencies during L2 naming on congruent trials compared to L1 naming (*t =* -2.29).

**Table 8:** Results on Naming latency in cued and voluntary block (Experiment 3).

	Cued block			Voluntary block			Across blocks		
Variable	Estimate	SE	*t*	Estimate	SE	*t*	Estimate	SE	*t*
Intercept	1000.12	75.2	13.3*	1663.78	85.17	19.53*	993.5	84.29	11.79*
Block							669.39	78.08	8.57*
Trial type	82.9	52.03	1.59	55.84	64.51	0.87	93.85	62.12	1.51
Congruency	106.59	52.25	2.04*	56.57	66.23	0.85	119.21	68.58	1.74^†^
Language	138.93	65.48	2.12*	46.94	66.03	0.71	135.71	69.65	1.95^†^
Trial type*Block							-45.95	75.31	-0.61
Language*Block							-75.97	77.94	-0.97
Congruency*Block							-50.27	82.86	-0.61
Trial type*Congruency	-132.65	72.44	-1.83^†^	51.03	99.67	0.51	-152.61	81.77	-1.89^†^
Congruency*Language	-125.61	69.99	-1.79^†^	-119.33	102.31	-1.17	-136.02	82.87	-1.64
Trial type*Language	-25.1	69.93	-0.36	-84.87	87.83	-0.97	-28.85	78.91	-0.37
Trial type*Congruency*Block							202.78	94.84	2.14*
Congruency*Languge*Block							25.57	95.18	0.27
Trial type*Language*Block							-50.34	90.45	-0.56
Trial type*Language*Congruency	120.58	99.75	1.21	165.8	149.49	1.11	142.37	91.54	1.55

* *p* < 0.05

^†^ *p* < 0.1.

#### Errors

Participants made more errors in naming when the language cue was incongruent with the cartoon's choice (ß = 0.56, SE = 0.21, *t =* 2.67) in the cued trials. However, congruency did not play a significant role in errors in the voluntary block (ß = 0.16, SE = 0.25, *t =* 0.65). None of the other main effects were significant in either the cued block or the voluntary block.

#### Correlational analysis

**Fig 6:**
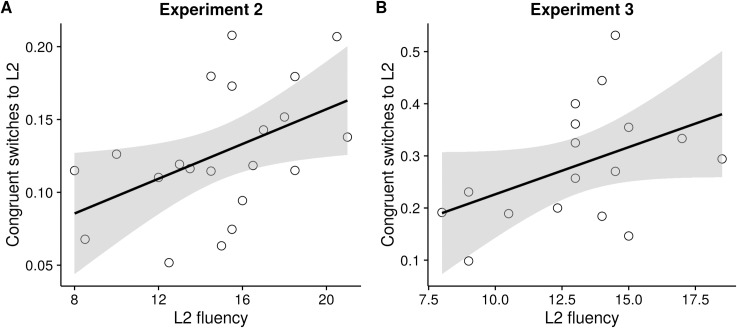
Correlation plot for L2 switch rate (when participants switched to the language indicated by the cartoon) vs L2 fluency for Experiment 2 and 3.

To examine the interaction between the language dominance of the participants and the cartoon's influence on switching, we examined correlations between switch rate and L2 fluency scores. For switch rate, we considered the percentage of switches to the language indicated by the cartoon (Fig 6). If speaker’s dominance modulated the influence of the cartoon, we should see a negative correlation between congruent L1 switches and L2 fluency. That is, participants highly dominant in English should resist the cartoon's influence to switch to L1. However, we did not find significant correlations between congruent L1 switches and L2 fluency in both Experiment 2 and 3. Interestingly, there was a positive correlation (*r*^2^ = 0.47, *p* = 0.03 in Expt. 2; *r*^2^ = 0.44, *p* = 0.06 in Expt. 3) between percentage of congruent L2 switches and L2 fluency.

### Discussion

The results showed that participants chose the language indicated by the cartoon more often than not, in spite of having a longer decision time. English was also chosen a higher number of times, replicating the results of Expt. 1 (“No constraint” instruction) and Experiment 2. Like in previous experiments, participants were not faster in naming when they went with the cartoon's language choice. Further, the correlational analysis revealed that participants who were highly dominant in L2 switched more to L2 when it was also chosen by the cartoon. This effect was found only in Experiment 2 and 3, but not in Experiment 1. Since participants chose the language before seeing the picture in Experiment 1, it is less likely in this case dominance interacted with the cartoon's influence in driving the switching behavior.

## General Discussion

In three experiments, we examined the influence of external, task-irrelevant cues in the form of a cartoon’s actions on choice during voluntary and cued naming in Hindi-English bilinguals. Speakers first saw a cartoon that waved at color squares linked to language cues before the production task. The cartoon’s actions were not relevant for the main experimental task. In all three experiments, a speaker’s choice of language was influenced by the color (mapped to a language) waved at by the cartoon. The cartoon’s choice of color also influenced speaker’s language switch pattern. This suggests that even external task-irrelevant cues in the environment linked to languages might influence voluntary choices related to object naming. Our results extend previous observations on voluntary naming studies [6],[7].

Cartoon’s waving at colored squares was random, but it also gave rise to a switching context. We expected speakers to be sensitive to this context in their switching behavior. Data from three experiments show that speakers also switched along with the cartoon. Switching was greater in the “Maintain balance” condition than in the “No- constraint” condition (Experiment 1). Thus, speakers' switching was influenced by their perception of the cartoon’s switching between colors. Gollan and Ferreira (2009) [6] had found higher switch rate when participants were forced to keep a balance (Experiment 2) indicating that the instruction to maintain balance modulated the top-down goal related to language planning of the speakers. It appears that while speakers may have their plans for switching between languages during free choice, the cartoon’s choice of color biased this decision in favor of one language. This influence also enhanced the repetition bias existing in the selection of responses.

Our data also suggests that the speaker’s language dominance might have interacted with cartoon’s influence on language switching. Gollan et. al. (2014) [7] examined voluntary switching as a function of language dominance of the participants and found that response accessibility in the dominant language was one of the driving factors for voluntary switching in bilinguals. The bilinguals studied in our experiments were dominant in English, as shown by higher semantic fluency scores on English across three experiments. We also observed that participants made fewer errors (Expt. 1 and 2) and were faster in naming in English (Expt. 3) compared to Hindi. Participants also chose English more often than Hindi in Experiment 2 and 3. No such effect was found in Experiment 1. Experiment 1 included the explicit choice stage where participants chose a language before seeing the pictures. We observed that English and Hindi were chosen equally often. In contrast, when participants had to choose the language after seeing the pictures, they chose English more. These results further support that idea that language dominance may modulate language selection, depending on when the choice is made. However, our results indicate that in spite of dominance level in a particular language, bilingual switching patterns were perhaps susceptible to external primes. This suggests a dynamic interaction between bottom-up and top-down forces during voluntary choices in action selection and execution. Further studies are needed to examine such interactions in-depth.

In all the three experiments, speakers were not necessarily faster when they undertook a choice similar to the cartoon. This suggests that on voluntary choice tasks, merely choosing an action does not guarantee that such a free choice facilitates the following action itself. From our results, it may seem that speakers may have been primed to select the language cue congruent with the cartoon’s choice, but this did not facilitate naming as such. It may also be that task choice and task performance in the linguistic context operate through different mechanisms. It is possible that contextual cues influence naming latencies in bilinguals only when the cues are relevant to their speech production.

We did not find faster naming latencies on voluntary trials compared to cued trials. This is in contrast to several non-linguistic switching studies where participants are found to be faster on voluntary trials [19],[20]. Thus, there seems to be a dissociation between choice and its influence on resulting actions. However, it is important to note that, Gollan et al. (2014) [7] had observed voluntary advantage on naming task only in the non-linguistic domain (Experiment 1). A similar advantage in linguistic naming was found when pictures to be named were repeated across trials (Experiment 2). Since we did not repeat pictures across trials, our results are consistent with Gollan et al. (2014) [7] (Experiment 1). Thus, it appears that linguistic and non-linguistic naming are different in terms of the mechanisms recruited especially in the voluntary scenario, as far as these data suggest.

Consistent with many previous studies, we found cued switch costs in all three experiments (significant in Expt. 1 and 3, descriptive differences in Expt. 2). However, the asymmetrical switch cost (that is, higher cost while switching from L2 to L1 as compared to switching from L1 to L2) was not observed. This asymmetry arises due to the greater amount of inhibition required to suppress a more dominant L1 while speaking in L2 [33]. Several studies have failed to observe this asymmetry [34],[35],[36],[37],[38], particularly during voluntary naming [8]. Since our participants were highly proficient in both their languages, it is possible that asymmetrical costs were not incurred during switching. Surprisingly, voluntary switch costs were not found consistently. Since we have no data to show what would have happened to the switching pattern without the cartoon, we cannot be sure if this result is due to the type of participants or because of the influence of the cartoon.

In our experiments, the participants were told that the cartoon was irrelevant to the main task, and thus they need not have followed the cartoon's choice of a color patch to choose a language for themselves. Nevertheless, this is a possible limitation of the study as the participants might have become aware of the fact that the cartoon's actions were directly connected to their choices. Therefore, it is possible that the participants might have realized that the colors indicated by the cartoon correspond to the color options given to them (for choosing a language). Also, we did not have other neutral colors (not mapped to any language) that might have disrupted such an explicit association. This limitation is especially valid for Experiment 1 (where participants chose the colors to indicate their choice of language before naming). In Experiment 2 and 3, participants did not explicitly choose a color to indicate language. Instead, they named the objects directly following the presentation of the cartoon. Thus, the participants may not have explicitly associated the cartoon's actions with their language choices. Nonetheless, to explore the implicit nature of external influences on language related decision making, such primes can perhaps be subliminal(We thank an annonymous reviewer for this suggestion). This can be examined in future studies.

Our experiments extend earlier studies on voluntary naming in many ways. Firstly, we have studied a distinct kind of bilingual population that have not been studied a lot. These bilinguals show dominance in L2 because of having acquired English early as L2. Secondly, we examined how external events influence voluntary choice. Previous studies by Gollan and colleagues have revealed how voluntary language switching is linked to language dominance and lexical accessibility via switch rate/cost. We did observe switch costs in three experiments but did not find asymmetry. However, the focus of our study was to examine the influence of external cues on language selection during voluntary naming. These experiments show that voluntary object naming is vulnerable to external influences. This provides evidence to the proposal that bilinguals who need to execute subtle control processes for conflict management might consider language cues in the environment during language selection and naming.

## Supporting Information

S1 Raw data Raw data for the dependent measures in Experiment 1. (XLSX)

S2 Raw data Raw data for the dependent measures in Experiment 2. (XLSX)

S3 Raw data Raw data for the dependent measures in Experiment 3. (XLSX)

S1 Appendix Line drawings used in the object naminf experiments. (DOC)

S2 Appendix Full structure of the models and the random effect variances derived from linear mixed effects analyses (Experiment 1–3). (DOC)
