# Supplementary material for: Task Irrelevant External Cues Can Influence Language Selection in Voluntary Object Naming: Evidence from Hindi-English Bilinguals
Source: PLoS One. 2017 Jan 12;12(1):e0169284. doi: 10.1371/journal.pone.0169284 (PMC5230772; doi:10.1371/journal.pone.0169284)
Supplement: S1 Appendix — (DOC) [file pone.0169284.s004.doc]

**Task irrelevant external cues can influence language selection in voluntary object naming: Evidence from Hindi-English bilinguals**

Divya Bhatia^1,*^, Seema Gorur Prasad^2^ , Kaushik Sake^2^ & Ramesh Kumar Mishra^2^

^1^Maharishi Dayanand University, Rohtak, India

^2^Center for Neural and Cognitive Sciences, University of Hyderabad, Hyderabad, India

**Corresponding author*

Divya Bhatia

Maharishi Dayanand University, Rohtak

Haryana, 124001

India

**email :** [**divyabhatia72@gmail.com**](mailto:divyabhatia72@gmail.com)

**S1 Appendix: Line drawings used in the object naming experiments (Experiment 1 - 3)**

**
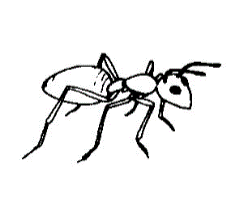

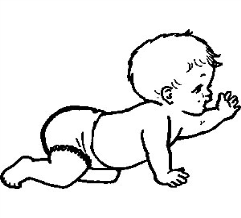

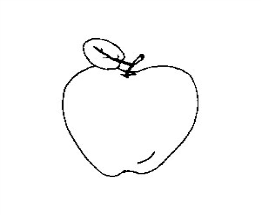

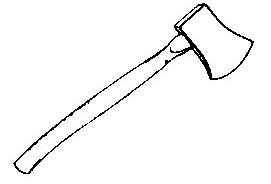
**

**
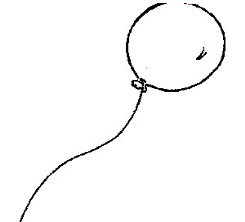
**

**English=Balloon**

**Hindi=Gubbara**

**English= Baby**

**Hindi=Bacha**

**English=Ant**

**Hindi=chinti**

**English=Axe**

**Hindi=Kulhadi**

**English=Apple**

**Hindi=Seb**


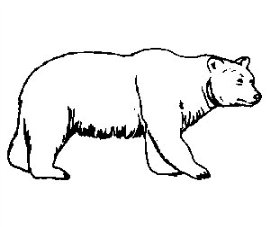

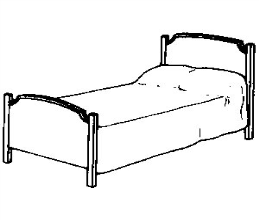

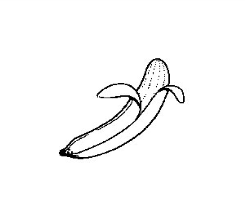

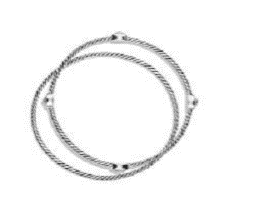

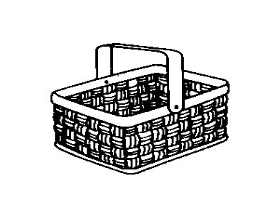


**English = Bed**

**Hindi=Bistar**

**English=Bear**

**Hindi=Bhalu**

**English=Basket**

**Hindi=Tokri**

**English= Bangle**

**Hindi=Chudi**

**English=Banana**

**Hindi=Kela**


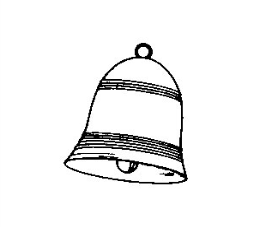

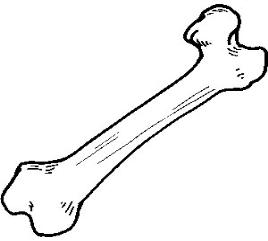

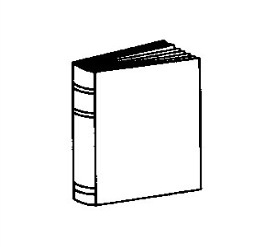

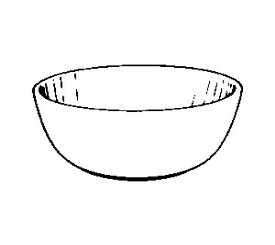

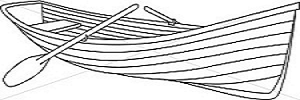


**English=Bowl**

**Hindi=Katora**

**English=Book**

**Hindi=Kitab**

**English=Bone**

**Hindi=Haddi**

**English=Boat**

**Hindi=Naav**

**English=Bell**

**Hindi=Ghanti**


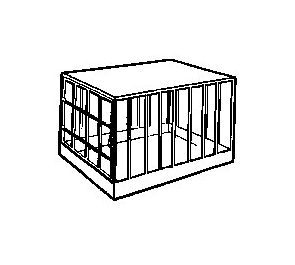

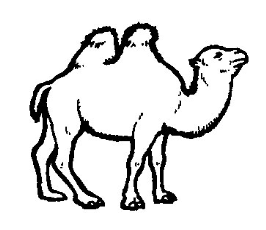


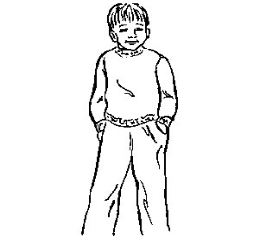

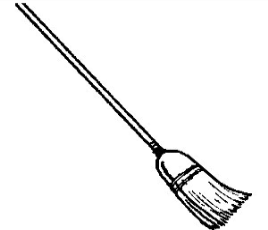

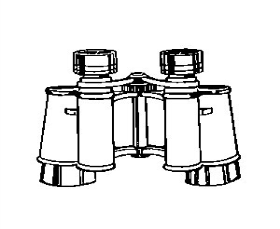


**English= Camel**

**Hindi= Oonth**

**English=Cage**

**Hindi=Pinjara**

**English=Broom**

**Hindi=Jharu**

**English=Binoculars**

**Hindi=Doorbeen**

**English=Boy**

**Hindi=Ladka**


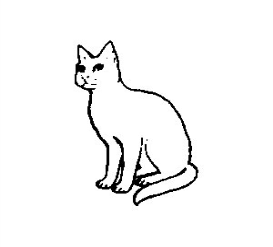

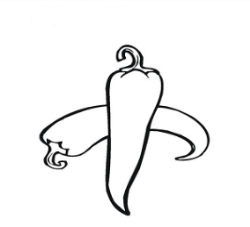

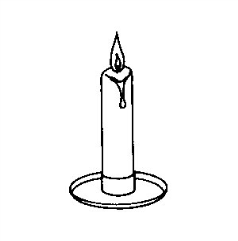

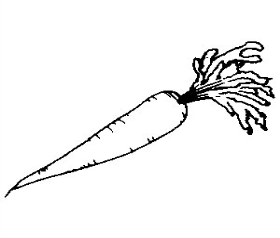

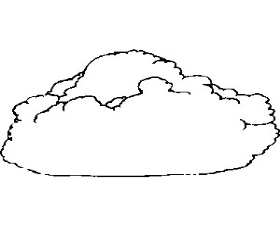


**English=Chilli**

**Hindi=Mirch**

**English=Cloud**

**Hindi=Baadal**

**English=Cat**

**Hindi=Billi**

**English=Carrot**

**Hindi=Gaajar**

**English=Candle**

**Hindi=Mombatti**


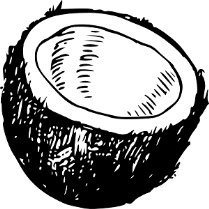

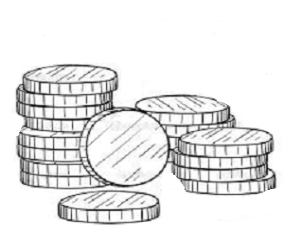

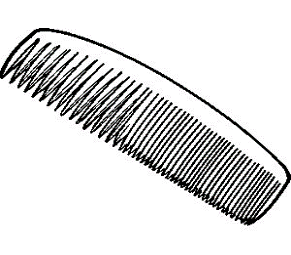

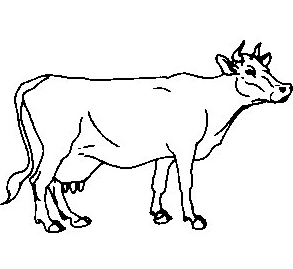

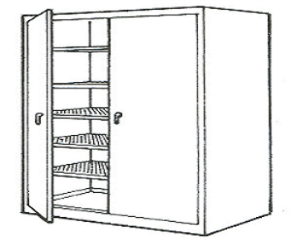


**English=Cupboard**

**Hindi=Almari**

**English=Comb**

**Hindi=Kangha**

**English=Cow**

**Hindi=Gaay**

**English=coins**

**Hindi=Sikke**

**English=Coconut**

**Hindi=Nariyal**


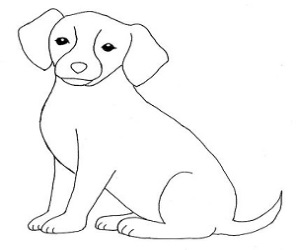

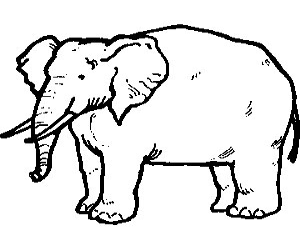

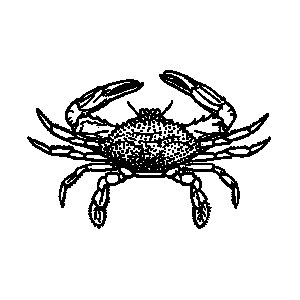

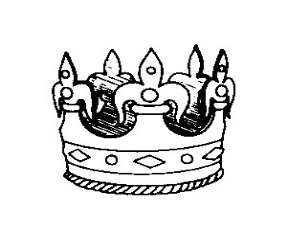

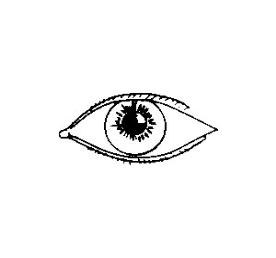


**English=Eye**

**Hindi=Ankh**

**English=Elephant**

**Hindi=Hathi**

**English=Dog**

**Hindi=Kutta**

**English=Crown**

**Hindi=Taaj**

**English=Crab**

**Hindi=Kekada**


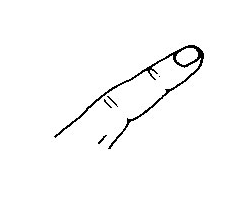

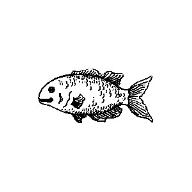

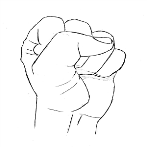

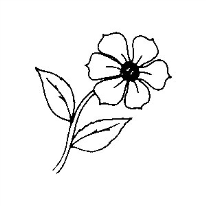

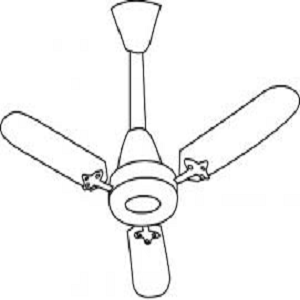


**English=Flower**

**Hindi=Phool**

**English=Fist**

**Hindi=Muthi**

**English=Fish**

**Hindi=Machli**

**English=Finger**

**Hindi=Anguli**

**English=Fan**

**Hindi=Pankha**


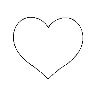

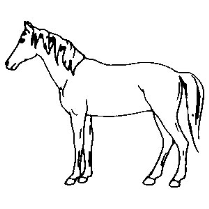

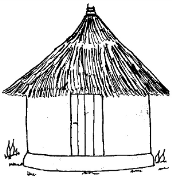

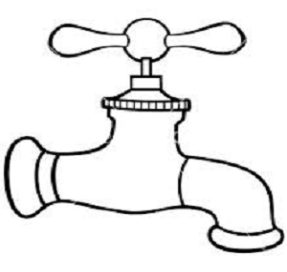

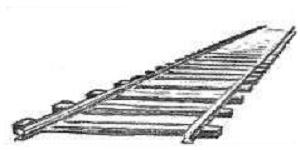


**English=Tap**

**Hindi=Nal**

**English=Hut**

**Hindi=Jhopdi**

**English=Horse**

**Hindi=Ghora**

**English=Heart**

**Hindi=Dil**

**English=Track**

**Hindi=Patri**


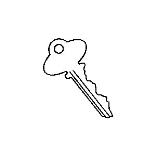

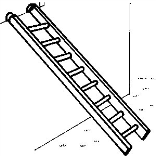


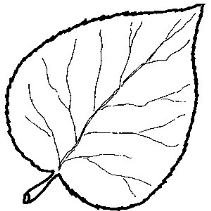

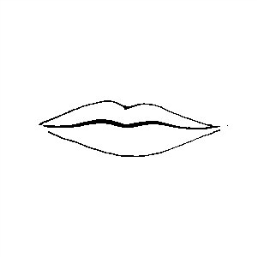

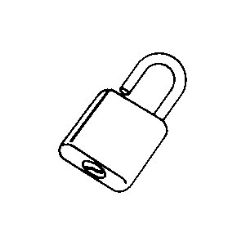


**English=Lock**

**Hindi=Tala**

**English=Lips**

**Hindi=Honth**

**English=Leaf**

**Hindi=Patta**

**English=Ladder**

**Hindi=Seedhi**

**English=Keys**

**Hindi=Chabi**


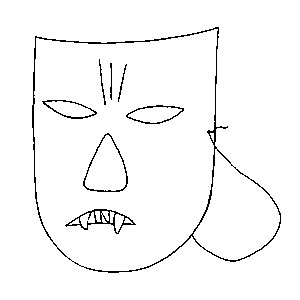

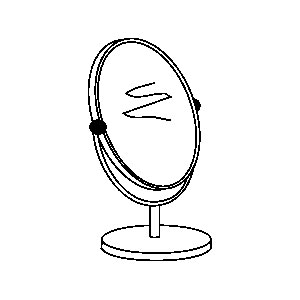


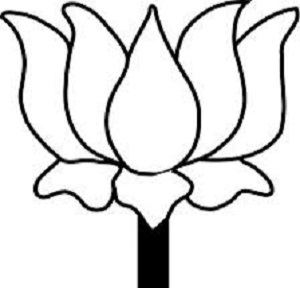

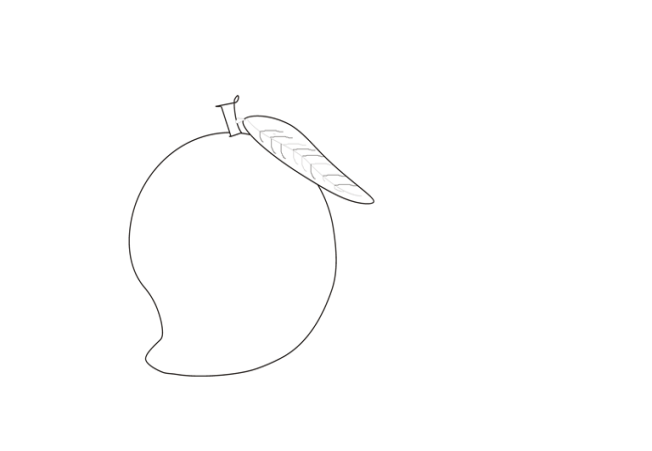

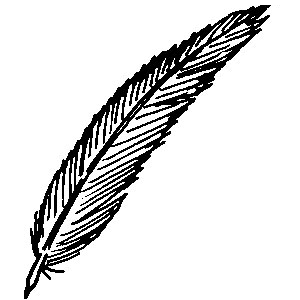


**English=Mirror**

**Hindi=Aina**

**English=Mask**

**Hindi=Mukhota**

**English=Mango**

**Hindi=Aam**

**English=Feather**

**Hindi=Pankh**

**English=Lotus**

**Hindi=Kamal**


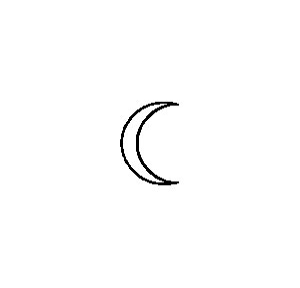

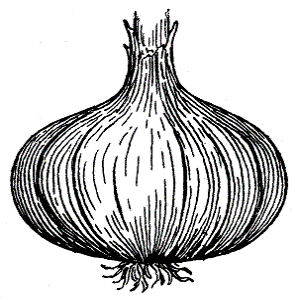

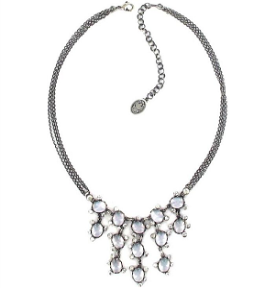

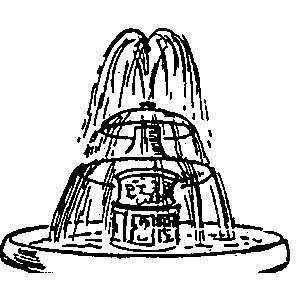

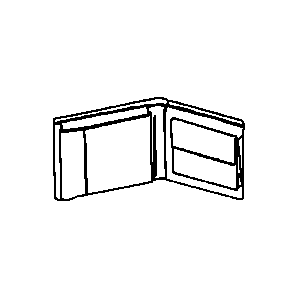


**English=Onion**

**Hindi=Payaj**

**English=Necklace**

**Hindi=Haar**

**English=Fountain**

**Hindi=Fuwara**

**English=Wallet**

**Hindi=Batua**

**English=Moon**

**Hindi=Chand**


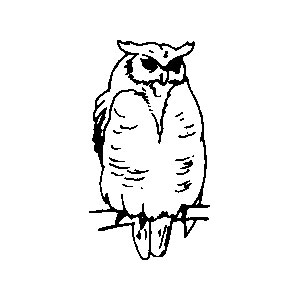

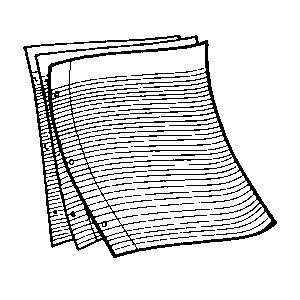

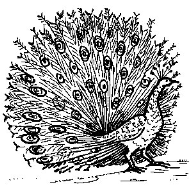

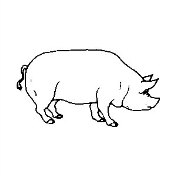

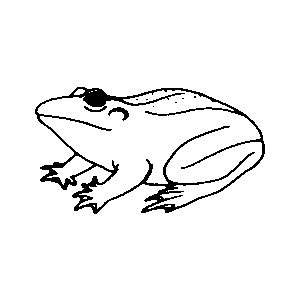


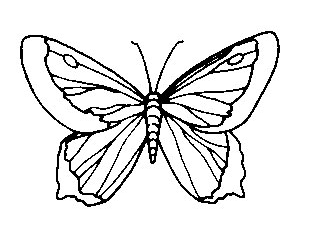

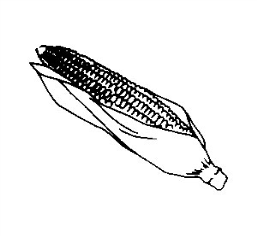

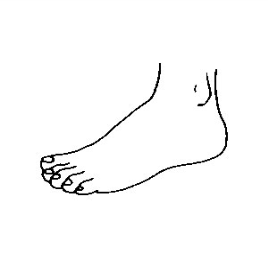

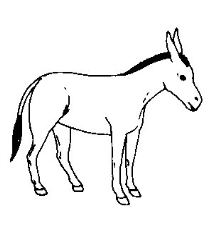

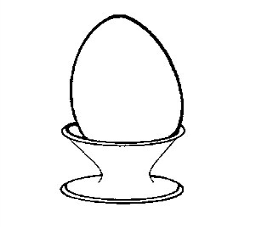


**English=Pig**

**Hindi=Suar**

**English=Peacock**

**Hindi=Mor**

**English=Frog**

**Hindi=Mendhak**

**English=Owl**

**Hindi=Ullu**

**English=Paper**

**Hindi=Kagaz**

**English=Butterfly**

**Hindi=Titli**

**English=Egg**

**Hindi=Anda**

**English=Donkey**

**Hindi=Gadha**

**English=Foot**

**Hindi=Per**

**English=Corn**

**Hindi=Makka**


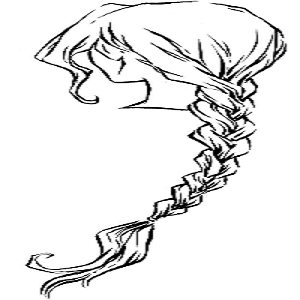

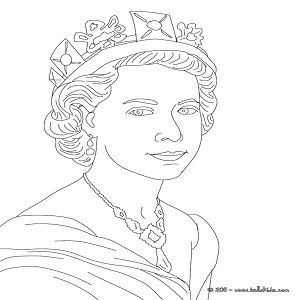

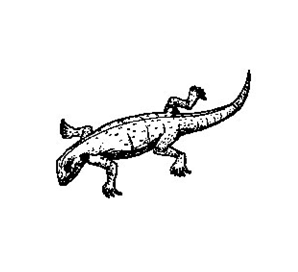

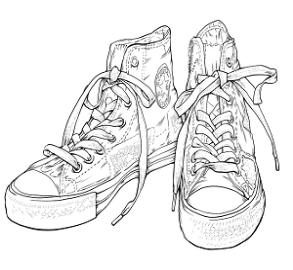

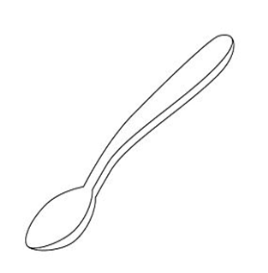


**English=Lizard**

**Hindi=Chipkali**

**English=Spoon**

**Hindi=Chamach**

**English=Plait**

**Hindi=Choti**

**English=Shoes**

**Hindi=Joote**

**English=Queen**

**Hindi=Rani**


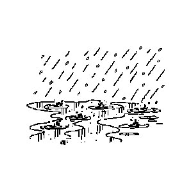

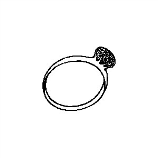

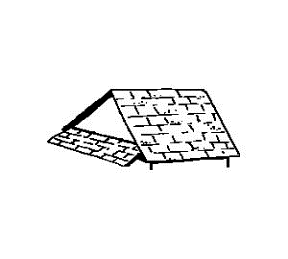

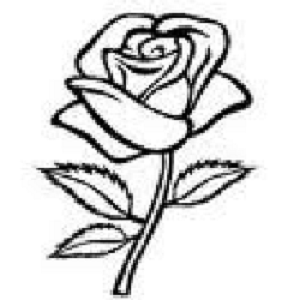

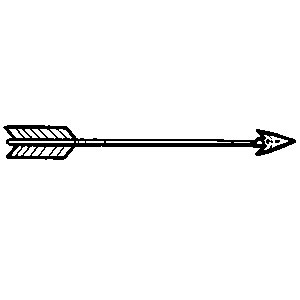


**English=Rose**

**Hindi=Gulab**

**English=Roof**

**Hindi=Chat**

**English=Arrow**

**Hindi=Teer**

**English=Ring**

**Hindi=Angoothi**

**English=Rain**

**Hindi=Barish**


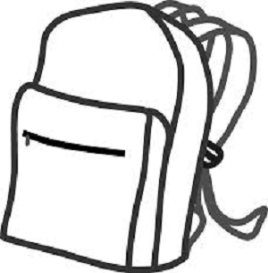

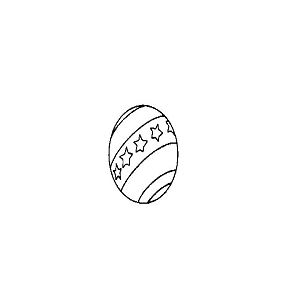


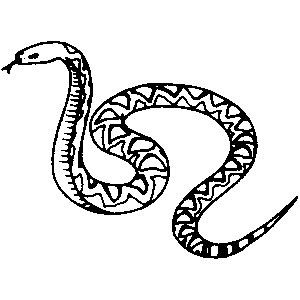

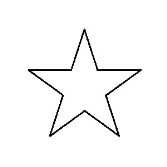

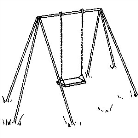


**English=Ball**

**Hindi=Gend**

**English=Bag**

**Hindi=Basta**

**English=Swing**

**Hindi=Jhula**

**English=Star**

**Hindi= Tara**

**English=Snake**

**Hindi=Saap**


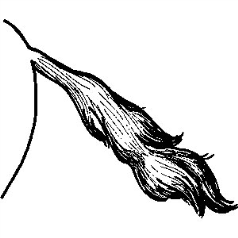

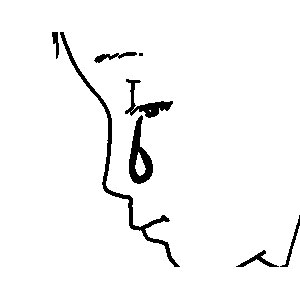

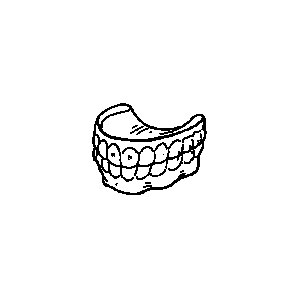

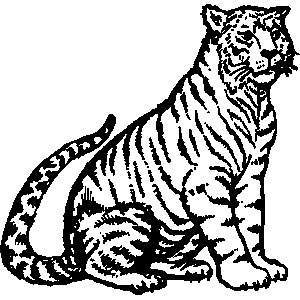

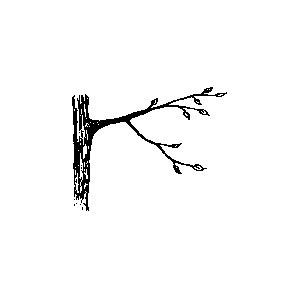


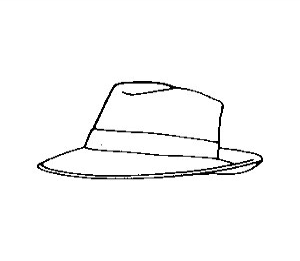

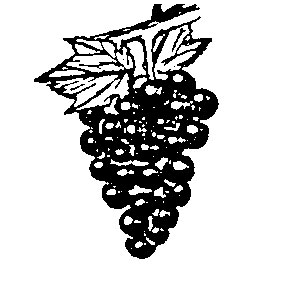

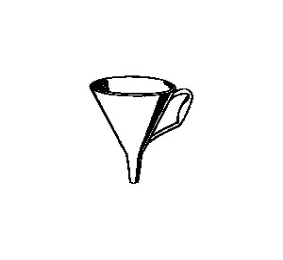

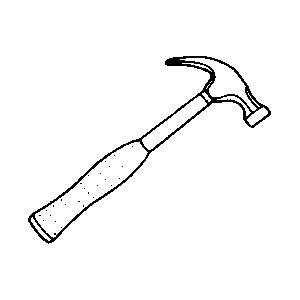

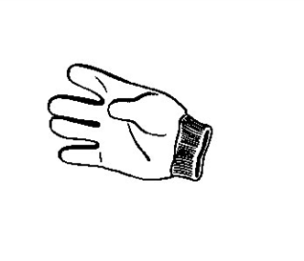


**English=Tiger**

**Hindi=Bagh**

**English=Branch**

**Hindi=Daali**

**English=Teeth**

**Hindi=Daanth**

**English=Tear**

**Hindi=Aansu**

**English=Tail**

**Hindi=Poonch**

**English=Funnel**

**Hindi=Keep**

**English=Grapes**

**Hindi=Angoor**

**English=Glove**

**Hindi=Dastana**

**English=Hat**

**Hindi=Topi**

**English=Hammer**

**Hindi=Hathoda**


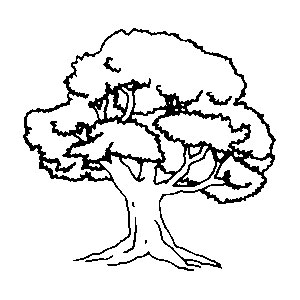

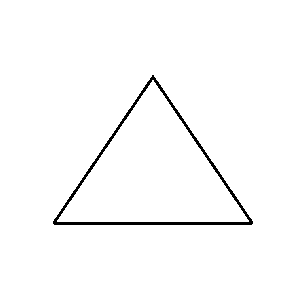

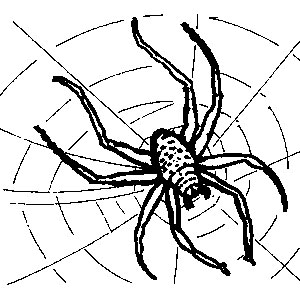

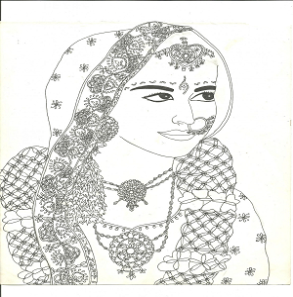

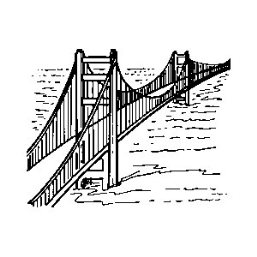


**English=Spider**

**Hindi=Makdi**

**English=Bridge**

**Hindi=Pul**

**English=Tree**

**Hindi=Ped**

**English=Triangle**

**Hindi=Trikon**

**English=Bride**

**Hindi=Dulhan**

**English=Flag**

**Hindi=Jhanda**

**English=Fire**

**Hindi=Aag**

**English=Nose**

**Hindi=Naak**

**English=Umbrella**

**Hindi=Chata**

**English=Ear**

**Hindi=Kaan**

**English=Clock**

**Hindi=Ghadi**

**English=Stairs**

**Hindi=Seedhi**

**English=Goat**

**Hindi=Bakri**

**English=Ghost**

**Hindi=Bhoot**

**English=Brinjal**

**Hindi=Baingan**

**English=Rat**

**Hindi=Chuha**

**English=Lion**

**Hindi=Sher**

**English=Scorpion**

**Hindi=Bichu**

**English=Ladyfinger**

**Hindi=Bhindi**

**English=Pot**

**Hindi=Matka**

**English=Diamond**

**Hindi=Heera**

**English=Doll**

**Hindi=Gudiya**

**English=Turtle**

**Hindi=Kachua**

**English=Thumb**

**Hindi=Angootha**

**English=Ship**

**Hindi=Jahaj**

**English=Knife**

**Hindi=Chaku**

**English=Kite**

**Hindi=Patang**

**English=Girl**

**Hindi=Ladki**

**English=King**

**Hindi=Raja**

**English=Duck**

**Hindi=Batakh**

**English=Lamp**

**Hindi=Batti**

**English=Nail**

**Hindi=Keel**

**English=Mosquito**

**Hindi=Machar**

**English=Needle**

**Hindi=Sooi**

**English=Monkey**

**Hindi=Bandar**

**English=Parrot**

**Hindi=Tota**

**English=Peas**

**Hindi=Matar**

**English=Rabbit**

**Hindi=Khargosh**

**English=Pillar**

**Hindi=Khamba**

**English=Nest**

**Hindi=Ghosla**

**English=Bat**

**Hindi=Balla**

**English=Scissors**

**Hindi=Kainchi**

**English=Skull**

**Hindi=Khopdi**

**English=Tomato**

**Hindi=Tamatar**

**English=Saturn**

**Hindi=Shani**

**English=Bow**

**Hindi=Dhanush**

**English=Box**

**Hindi=Dibba**

**English=Chess**

**Hindi=Shatranj**

**English=Carpet**

**Hindi=Dari**

**English=Cook**

**Hindi=Bawarchi**

**English=Ear-ring**

**Hindi=Kundal**

**English=Deer**

**Hindi=Hiran**

**English=Door**

**Hindi=Darwaza**

**English=Farmer**

**Hindi=Kisaan**

**English=Cucumber**

**Hindi=Kheera**

**English=Handcuffs**

**Hindi=Hathkadi**

**English=Kettle**

**Hindi=Ketli**

**English=Gun**

**Hindi=Bandook**

**English=Papaya**

**Hindi=Papita**

**English=Knot**

**Hindi=Gaanth**

**English=Pear**

**Hindi=Nashpati**

**English=Roots**

**Hindi=Jud**

**English=Soldier**

**Hindi=Sainik**

**English=Road**

**Hindi=Sadak**

**English=Squirrel**

**Hindi=Gilhari**
