# Supplementary material for: Task Irrelevant External Cues Can Influence Language Selection in Voluntary Object Naming: Evidence from Hindi-English Bilinguals
Source: PLoS One. 2017 Jan 12;12(1):e0169284. doi: 10.1371/journal.pone.0169284 (PMC5230772; doi:10.1371/journal.pone.0169284)
Supplement: S2 Appendix — (DOC) [file pone.0169284.s005.doc]

**Task irrelevant external cues can influence language selection in voluntary object naming: Evidence from Hindi-English bilinguals**

Divya Bhatia^1,*^, Seema Gorur Prasad^2^ , Kaushik Sake^2^ & Ramesh Kumar Mishra^2^

^1^Maharishi Dayanand University, Rohtak, India

^2^Center for Neural and Cognitive Sciences, University of Hyderabad, Hyderabad, India

**Corresponding author*

Divya Bhatia

Maharishi Dayanand University, Rohtak

Haryana, 124001

India

**email :** [**divyabhatia72@gmail.com**](mailto:divyabhatia72@gmail.com)

**S2 Appendix: Linear Mixed effects analyses**

Following the presentation of results in Gambi and Hartsuiker (2016), we report below the random

effect variances for the fixed effects in all the analyses (Experiment 1 – 3). (S) refers to variance due to the random effect of subjects, (I) refers to item-wise random effect variance. Estimates and standard errors have been reported in the text and the table numbers are given here for reference.

Table A. Analysis on choices (Experiment 1; Refer to Table 2 in the text).

| Variable | “Maintain balance” instruction | “No constraint” | Across instructions |
| --- | --- | --- | --- |
|  |  |  |  |
| Intercept | 0 (S) | 7.726e-10 (S) | 1.852e-09 (S) |
| Instruction | NA | NA | 2.802e-02 (S) |
| Trial type | 2.816e-01 (S) | 1.667e-01 (S) | 6.945e-02 (S) |
| Congruency | 3.207e-03 (S) | 7.131e-02 (S) | 2.602e-02 (S) |
| Language | 1.762e-09 (S) | 1.558e-01 (S) | 4.930e-02 (S) |
| Instruction * Trial type | - | - | - |
| Instruction*Congruency | - | - | - |
| Instruction * Language | - | - | - |
| Congruency*Trial type | - | - | - |
| Language*Trial Type | - | - | - |
| Congruency* Language | - | - | - |
| Congruency*Language*Instruction | - | - | - |

Note: Subject-wise random effect variances are presented (for all experiments) as the analysis on choices included only subjects as random effects, but not items.

Table B: Analysis on naming latency (Experiment 1: Cued block; Refer to Table 3 in the text)

| Variable | Random effect variance |
| --- | --- |
| Intercept | 13090 (S); 11350 (I) |
| Trial type | 1.069e-09 (S); 3164 (I) |
| Congruency | 0 (S); 1.230e-09 |
| Language | 760 (S); 1542 (I) |
| Congruency*Trial type | - |
| Language*Trial Type | - |
| Congruency*Language | - |
| Congruency*Lamguage*Trial type | - |

Table C: Analysis on naming latency (Experiment 1: Voluntary block; Refer to Table 4 in the text)

| Variable | “Maintain balance” instruction | “No constraint” | Across instructions |
| --- | --- | --- | --- |
|  |  |  |  |
| Intercept | 29502 (S); 10629 (I) | 21471 (S); 7039 (I) | 5690 (S); 9054 (I) |
| Instruction | NA | NA | - |
| Trial type | 812 (S); 651 (I) | 3652 (S); 2469 (I) | 6497 (S); 3321 (I) |
| Congruency | 834 (S); 2104 (I) | 1525 (S); 1864 (I) | 2030 (S); 1248 (I) |
| Language | 986 (S); 4079 (I) | 5118 (S); 6459 (I) | 3876 (S); 4415 (I) |
| Instruction * Trial type | - | - | - |
| Instruction*Congruency | - | - | - |
| Instruction * Language | - | - | - |
| Congruency*Trial type | - | - | - |
| Language*Trial Type | - | - | - |
| Congruency* Language | - | - | - |
| Congruency*Language*Instruction | - | - | - |

Table D: Analysis on choices (Experiment 2; Refer to Table 5 in the text)

| Variable | Random effect variance |
| --- | --- |
| Intercept | 0.01527 (S) |
| Trial type | 0.08530 (S) |
| Congruency | - |
| Language | 0.04770 (S) |
| Congruency*Trial type | - |
| Language*Trial Type | - |
| Congruency*Language | - |
| Trial*Congruency*Language | - |

Table E: Analysis on naming latency (Experiment 2: Cued and voluntary block; Refer to Table 6 in the text)

| Variable | Cued block | Voluntary block | Across blocks |
| --- | --- | --- | --- |
|  |  |  |  |
| Intercept | 26820 (S); 7375 (I) | 41790 (S);5173 (I) | 25256 (S); 5201 (I) |
| Block | NA | NA | 9624 (S) |
| Trial type | 317 (S); 3199 (I) | 4.022e-10 (S); 3241 (I) | 140 (S); 3410 (I) |
| Congruency | 9.838e-10 (S) | 9.224e-09 (S) | 1560 (I) |
| Language | 4249 (S); 1613 (I) | 0 (S); 7.618e-11 | - |
| Trial type*Block | - | - | - |
| Language*Block | - | - | - |
| Congruency*Block | - | - | - |
| Trial type*Congruency | - | - | - |
| Congruency*Language | - | - | - |
| Trial type*Language | - | - | - |
| Trial type*Congruency*Block | - | - | - |
| Congruency*Languge*Block | - | - | - |
| Trial type*Language*Block | - | - | - |
| Trial type*Language*  Congruency | - | - | - |

Table F: Analysis on choices (Experiment 3; Refer to Table 7 in the text)

| Variable | Random effect variance |
| --- | --- |
| Intercept | 2.537e-09 (S) |
| Trial type | 3.305e-02 (S) |
| Congruency | - |
| Language | 2.723e-02 (S) |
| Congruency*Trial type | - |
| Language*Trial Type | - |
| Congruency*Language | - |
| Trial*Congruency*Language | - |

Table G: Analysis on naming latency (Experiment 3: Cued and voluntary block; Refer to Table 8 in the text)

| Variable | Cued block | Voluntary block | Across blocks |
| --- | --- | --- | --- |
|  |  |  |  |
| Intercept | 76430 (S); 7666 (I) | 112977 (S); 22487 (I) | 67985 (S); 16736 (I) |
| Block | NA | NA | 28070 (S) |
| Trial type | 2211 (S); 5868 (I) | 4282 (S); 8105 (I) | 2243 (S); 6349 (I) |
| Congruency | 4.711e-09 (S) | 1103 (S); 0 (I) | 1208 (S); 0 (I) |
| Language | 21020 (S); 0 (I) | 1132 (S); 15988 (I) | 10298 (S); 6652 (I) |
| Trial type*Block | - | - | - |
| Language*Block | - | - | - |
| Congruency*Block | - | - | - |
| Trial type*Congruency | - | - | - |
| Congruency*Language | - | - | - |
| Trial type*Language | - | - | - |
| Trial type*Congruency*Block | - | - | - |
| Congruency*Languge*Block | - | - | - |
| Trial type*Language*Block | - | - | - |
| Trial type*Language*  Congruency | - | - | - |
